# A multilevel exploration of *Avena strigosa* diversity as a prelude to promote alternative crop

**DOI:** 10.1186/s12870-019-1819-6

**Published:** 2019-07-03

**Authors:** Wiesław Podyma, Paulina Bolc, Joanna Nocen, Marta Puchta, Sylwia Wlodarczyk, Boguslaw Lapinski, Maja Boczkowska

**Affiliations:** 1 Plant Breeding and Acclimatization Institute (IHAR) - National Research Institute, Radzików, Poland; 2 Polish Academy of Sciences Botanical Garden – Center for Biological Diversity Conservation in Powsin, Warszawa, Poland

**Keywords:** Alternative crop, *Avena strigosa*, Diversity, Generalized Procrustes analysis, Germplasm, Isoenzymes, Joint analysis, Morphology, Sand oat, SRAP

## Abstract

**Background:**

Sand oat (*Avena strigosa* Schreb.), one of the four cultivated species of the genus *Avena,* could be considered as another alternative crop. In gene banks 865 germplasm samples of this species have been preserved that have not been thoroughly investigated so far. The results of phenotyping (36 traits), isoenzymatic (12 systems) and genetic (8 pairs of Sequence-related amplified polymorphism markers) variation were used to obtain the complete description of 56 accessions diversity originated from different parts of world.

**Results:**

Breeded and weedy forms represented similar pool of morphological traits that indicated a short-term and extensive breeding process, albeit all accessions which we classified as cultivated were characterized by better grain and green mass parameters compared to the weedy ones. Isoenzymes showed relationships with geographical origin, which was not possible to detect by SRAP markers. There was no similarity between morphological and biochemical results. The polymorphism level of SRAP markers was lower than indicated by the available literature data for other species, however it may result from the analysis of pooled samples of accessions with a high internal variability. The extensive type of breeding and its relatively short duration was also reflected in the population structure results. Joint analysis revealed that a secondary centre of diversity is being created in South America and that it has its genealogy from the Iberian Peninsula.

**Conclusions:**

Despite the relatively large representation of this species is in various gene banks, it is highly probable that the vast majority of stored worldwide accessions are duplicates, and the protected gene pool is relatively narrow. Sand oat meets all the requirements for an alternative crop species, but further studies are needed to identify the genotypes/populations with the most favourable distribution of utility and quality parameters.

## Background

Global climate change implicates search for alternative crops that will be more stable in yielding in conditions of increased temperature and drought stress, will also be less resource-dependent, resistant to pathogens and pests, and will be rich in nutrients. One of these alternative crops may become sand oat (*Avena strigosa* Schreb.). It is one of the four cultivated species of the genus *Avena* besides *A. sativa L.*, *A. byzantina* and *A. abyssinica.* From the economical point of view, the most important is common oat (*A. sativa*) cultivated for grain while other species are marginal. Sand oat (*A. strigosa*) is currently grown only in South America as a winter season cover crop and as forage and pasture in the Southwestern U.S. [1]. In the past, sand oat was more important as a grain crop. Until the seventeenth century it was the main oat species grown on British Islands. Until the beginning of the twentieth century it was cultivated on poor soils in Scotland, in the Iberian Peninsula and in numerous European countries [2]. Sand oat is also a carrier of resistance genes for *Ustilago avenae* (Pers.) Rostr*.*, *Puccinia graminis f. sp. avenae* Erikss. & Henning and *Puccinia coronata f. sp. avenae* P. Syd. & Syd [3–6]. Grain of this species has a high nutritional value, i.e. it contains 27–52% more protein, 14–27% more fat and 38–72% more polysaccharides than common oat [7]. Literature showes also information about health promoting ingredients such as β-glucan, tocopherols, tocotrienols, phenolic alkaloids and phenolic acids. However, the content of these compounds is very variable and may depend on both genetic and environmental factors [7]. Therefore, this species should be subject to more extensive research in terms of both biochemical and genetic differentiation. Sand oat is described as an anthropogenic archaeophyte that is absent in natural and semi-natural habitats. Therefore, a trend towards extinction along with the cessation of its cultivation was observed and in the 1980s it was even claimed to be threatened with extinction [2, 8, 9]. According to the IUCN Red List, the current knowlage on the abundance of this species is insufficient for determining the degree of extinction threatand therefore it is globally assessed as Data Deficient (DD) with unknown current population trend [10]. Currently 845 accessions are preserved in gene banks worldwide, but there is high probability that a large part of them are duplicates deposited in various institutions [11]. Genetic resources of this species have not been extensively studied so far [8, 12–15]. The characterisation of the diversity preserved in the gene bank collections is essential for their effective conservation and utilization. Fundamentally, it involves description of morphological and agromorphological traits. However, the information provided by them is often very limited and can be influenced by environmental variation. This limitation can be overcome by biochemical techniques such as isozymes or molecular techniques that analyse DNA polymorphism. Isozymes are multiple enzyme forms in a single species. They catalyse the same reaction but may differ in various ways. They could be a genetically independent proteins or genetic variants (allozymes), conjugated or derived proteins, polymers of a single subunit etc. They are not subject to environmental influences, inherited as simple Mendelian units and are codominant in nature. However, their level of polymorphism is quite limited, and the methodology of their analysis is complex. Currently, isozymes play minor role in diversity analysis, they were replaced by molecular DNA techniques, but in literature some previous data of sand oat diversity can be found [8]. Sequence-related amplified polymorphism (SRAP), developed by Li and Quiros [16], is one of numerous techniques based on nucleic acid polymorphism. It is a simple, inexpensive, reproducible, versatile and effective technique for producing genome-wide DNA fragments. This PCR-based marker system targets coding regions without prior knowledge of its sequence by targeting the forward primer at GC-rich exons and revers primer at AT-rich promoters, introns and spacers [16]. Primers are 18 (forward) and 17 (reverse) nucleotide long and they consist of the following parts: the first 11 or 10 bases at the 5′-end are a ‘filer’ sequence with no specific composition. They are followed by CCGG (forward) or AATT (reverse) and finally, at the 3′-end there are three selective nucleotides [16]. Studies of 15 biotypes of *Buchloe dactyloides* (Nutt.) Englem. provided more information than RAPD, ISSR and SSR markers [17]. They were applied both in studies at the level of inter- and intraspecies systematics [18]. They were also used to characterize the diversity of genetic resources of such species as *Cucumis melo* L, *Cucurbita maxima* Duchesne, *Cucurbita moschata* Duchesne, *Cucurbita pepo* L., *Solanum tuberosum* L. and *Citrus* and its relative species [19–24]. SRAP markers have never been used for *A. strigosa* genetic diversity analysis.

In this study morphological traits, isozymes and SRAP (Sequence-related amplified polymorphism) markers, were used in integrative research of sand oat (*A. strigosa*) germplasm variation. The objective was to obtain the complete description of diversity of accessions originated from different parts of world.

## Results

Basing on the botanical diversity, seven botanical varieties of sand oat were identified within the set of accessions (*albida* Marq., *strigose* Rod et Sold., *gilva* Mordv., *melanocarpa* Mordv., *intermedia* Marq., *nigra* Marq., and *fusca* Marq.). Within each accession from one to four varieties were recorded. Of 56 accessions 16 belonged to one botanical variety, 30 to two, nine to three and one to four botanical varieties. This indicated the internal heterogeneity of the material (Table 1). Within the set of botanical varieties occurred with the following frequency: var. *albida* in one accession, var*. strigosa* in 51 accessions, var. *gilva* in 40 accessions, var. *melanocarpa* in six accessions, var. *intermedia* in seven accessions and var. *fusca* in one accession.

**Table 1:** The list of surveyed accessions. The accessions obtained from long term storage of NCPGR supplemented with origin data, other databases records, improvement status, botanical identification and isoenzymatic profile

No.	Accession number	Country of origin	Year of acquisition	Longitude	Latitude	Altitude	Other accession numbers	Improvement status	Botanical variety	Isoenzymatic profile
1	50880	Poland (POL)	1939	50 00 00 N	20 00 00 E	173	PI 131641; CI 3815	*BR*	*2, 3, 4*	*U*
2	50993	Germany (DEU)	1977	54 30 00 N	9 19 60 E	4		*CU*	*2, 3*	*U*
3	51000	Portugal (PRT)	na	39 47 31 N	9 06 28 W	39	AVE 3966	*CU*	*3*	*U*
4	51022	Brasil (BRA)	1940	10 00 00 S	55 00 00 W	320		*CU*	*2, 3*	*U*
5	51105	France (FRA)	1919	46 00 00 N	2 00 00 E	375	VIR 2172	*CU*	*2, 6*	*C*
6	51149	Brasil (BRA)	1945	30 00 00 S	53 00 00 W	320	CIav 4639; CI 4639	*CU*	*2, 3*	*U*
7	51150	United Kingdom (GBR)	1988	52 24 54 N	4 59 21 W	138		*CU*	*2, 3, 4*	*B*
8	51199	Bulgaria (BGR)	1970	43 00 00 N	25 00 00 E	472	CIav 9012; CN 3066	*CU*	*2, 3, 6*	*A*
9	51499	former Soviet Union (SUN)	1988	56 00 00 N	38 00 00 E	180		*CU*	*2, 3*	*C*
10	51518	Poland (POL)	1988	51 59 00 N	21 41 00 E	140		*WE*	*2*	*C*
11	51520	Poland (POL)	1988	51 56 00 N	22 23 00 E	150		*WE*	*2*	*A*
12	51523	Poland (POL)	1988	51 37 00 N	21 59 00 E	140		*WE*	*2, 3*	*U*
13	51524	Poland (POL)	1988	51 37 00 N	21 59 00 E	140		*WE*	*2, 3*	*B*
14	51574	Uruguay (URY)	1970	34 20 00 S	57 43 00 W	85	CN 3068	*BR*	*2, 3*	*B*
15	51575	Netherlands (NLD)	1970	52 30 00 N	5 45 00 E	30	CN 3070; CI 9022; CD 3916	*BR*	*2, 3*	*A*
16	51578	Uruguay (URY)	1951	34 49 59 S	56 10 00 W	43	PI 194201	*BR*	*2, 3*	*D*
17	51579	Ukraine (SUN)	1940	48 18 00 N	25 55 59 E	228	PI 258727, 9285	*CU*	*2*	*A*
18	51580	Lithuania (SUN)	1917	56 00 00 N	24 00 00 E	77	PI 258728, 2168	*CU*	*2, 9*	*B*
19	51582	Spain (ESP)	1927	43 00 00 N	7 34 00 W	465	PI 258730, VIR 5199	*CU*	*2, 3*	*C*
20	51583	Spain (ESP)	1927	43 00 00 N	7 34 00 W	465	PI 258731, VIR 5201	*CU*	*2, 3*	*C*
21	51584	France (FRA)	1919	46 00 00 N	2 00 00 E	375	PI 258732, VIR 2172	*CU*	*3*	*C*
22	51585	Poland (POL)	1961	50 00 00 N	20 00 00 E	173	PI 274609	*BR*	*6*	*U*
23	51586	Poland (POL)	1961	50 00 00 N	20 00 00 E	173	PI 274610	*BR*	*2*	*B*
24	51596	Chile (CHI)	1979	37 31 59 S	72 19 00 W	150	PI 436103; CN 81758	*WE*	*2, 3, 4*	*D*
25	51597	Chile (CHI)	1979	39 01 52 S	72 52 00 W	250	PI 436105, CN 81760	*WE*	*2, 3, 4*	*D*
26	51598	Poland (POL)	1988	52 27 00 N	21 28 59 E	100		*WE*	*2, 3*	*B*
27	51613	Poland (POL)	1988	52 24 40 N	21 43 44 E	120		*WE*	*2, 3*	*B*
28	51730	Brasil (BRA)	1940	10 00 00 S	55 00 00 W	320		*CU*	*2, 3*	*D*
29	51731	United Kingdom (GBR)	1920	52 24 54 N	4 59 21 W	138		*CU*	*2, 3*	*U*
30	51733	Spain (ESP)	1990	40 00 00 N	4 00 00 W		CN 21995; CAV 2838; Cc 7062	*WE*	*2, 3*	*A*
31	51734	Slovakia (SVK)	1977	48 52 49 N	22 18 04 E	410	AVE 1714	*WE*	*2*	*B*
32	51735	Slovakia (SVK)	1972	48 34 00 N	19 36 00 E	842	AVE 2558	*WE*	*2, 3*	*A*
33	51736	Slovakia (SVK)	1974	48 34 00 N	19 50 00 E	330	AVE 1438	*WE*	*2, 3*	*A*
34	51737	Slovakia (SVK)	1974	48 34 00 N	19 50 00 E	330	AVE 1467	*WE*	*2, 3*	*A*
35	51738	Germany (DEU)	1917	54 32 00 N	10 13 00 E	7	AVE 415	*BR*	*2, 3*	*A*
36	51739	Germany (DEU)	1917	54 32 00 N	10 13 00 E	7	AVE 414	*BR*	*2, 3*	*B*
37	51740	Spain (ESP)	1978	37 34 00 N	6 45 00 W	260	AVE 1867	*WE*	*2, 3*	*A*
38	51741	Spain (ESP)	1965	28 28 58 N	16 20 30 W	630	AVE 1129	*WE*	*2, 3*	*C*
39	51742	Spain (ESP)	1978	39 27 00 N	5 19 00 W	10	AVE 1874	*WE*	*6*	*D*
40	51743	Spain (ESP)	1978	39 20 12 N	5 29 32 W	640	AVE 1873	*WE*	*2, 3, 4*	*D*
41	51744	Spain (ESP)	1979	40 20 12 N	6 29 32 W	640	AVE 1898	*WE*	*1*	*U*
42	51745	Ethiopia (ETH)	1952	8 00 00 N	38 00 00 E	1330	AVE 488	*WE*	*2, 3, 4*	*C*
43	51746	unknown	na	na	na	na		na	*2, 3*	*U*
44	51747	United Kingdom (GBR)	na	52 24 54 N	4 59 21 W	138	Cc 4093	*BR*	*2, 3, 6, 7*	*U*
45	51748	United Kingdom (GBR)	1976	52 24 54 N	4 59 21 W	138	03C0701091; Cc 4659	*BR*	*2*	*B*
46	51749	France (FRA)	1919	46 00 00 N	2 00 00 E	375	AVE 1111, VIR 2172	*CU*	*2, 3, 6*	*A*
47	51751	Poland (POL)	1976	49 40 40 N	22 30 03 E	361		*WE*	*2*	*U*
48	51752	Poland (POL)	1980	53 37 48 N	23 09 12 E	150		*WE*	*2, 3*	*U*
49	51753	Poland (POL)	1976	50 18 00 N	21 45 00 E	220		*WE*	*2*	*B*
50	51754	Poland (POL)	1980	53 37 48 N	23 09 12 E	150		*WE*	*2*	*A*
51	51755	Poland (POL)	1986	51 21 25 N	21 35 02 E	157		*WE*	*2, 3*	*A*
52	51756	Poland (POL)	1976	50 18 47 N	21 50 16 E	185		*WE*	*2*	*A*
53	51757	Portugal (PRT)	na	39 30 00 N	8 00 00 W	372		*BR*	*2, 3*	*D*
54	51758	Germany (DEU)	before 1945	52 13 40 N	11 00 35 E	136	AVE35	*CU*	*2, 3*	*A*
55	51759	Portugal (PRT)	na	39 30 00 N	8 00 00 W	372		*BR*	*2, 6*	*A*
56	51760	Poland (POL)	1986	49 31 00 N	19 47 00 E	765		*WE*	*2, 3*	*A*

Improvement status: *BR* Breeding/research material, *CU* Cultivar, *W* Weedy; Botanical variety: 1 – var. *albida,;* 2 – var. *strigosa,* 3 – var. *gilva,* 4 - var. *melanocarpa,;* 6 – var. *intermedia,* 7 – var. *nigra,* 9 – var. *fusca;* Isoenzymatic profile: *A, B, C* and *D* – homogeneous groups; *U* – unique profile

The accessions collected in Spain were botanically the most diverse and contained five out of seven botanical varieties identified in the set. Two accessions were pure var. *albida* (PL 51744_(41)_) and var. *intermedia* (PL 51742_(39)_). Five accessions were a mixture of var. s*trigosa* and *gilva* and one additionally contained individuals of var. *melanocarpa.* Three botanical varieties: s*trigosa, gilva* and *intermedia* were identified in Portuguese accessions. The same three varieties was found within French accessions. They differed from each other in composition although, as indicated passport data, all three were derived from one accession VIR 2172 gathered by N.I. Vavilov in 1919 and preserved in N.I. Vavilov Institute of Plant Genetic Resources in St. Petersburg. The accessions obtained from The United Kingdom contained four botanical varieties s*trigosa, gilva, melanocarpa* and *intermedia* but within accession compositions were diverse. Among 16 Polish accessions as many as seven was homogeneous var. s*trigosa* and one *intermedia.* The mixture of var. s*trigosa* and var. *gilva* was identified in seven accessions and in one more var. *melanocarpa* was admixed. South American accessions originated from Brazil and Uruguay were composed of two botanical varieties i.e. s*trigosa* and *gilva* whereas Chilean ones contained addition of var. *melanocarpa* individuals. For more details see Table 1.

### Morphology

More than half of the analysed accessions were mixtures of grains with grey and brown lemma (55%). In six accessions beside grey and brown lemma an admixture of grains with black lemma was observed. Fourteen accessions have only grains with grey lemma. Among them, ten were homogenous variety *strigosa* and two *intermedia.* Only one accession i.e. PL 51744_(41)_ had grains with white lemma.

In general, South American plants had short awns and high weight of thousand grains, the Spanish accessions had short and narrow flag leaves while the Polish ones were short with short upper internodes and had long rachillas and glumes. Cultivated accessions and breeding or research materials were higher and had longer upper internodes than weedy ones. They also had high mean number of spikelets, short rachillas and glumes. Weedy plants were short and have short upper internodes.

The morphological diversity of tested set of sand oat was at the moderate-low level (Table 2). Among quantitative traits it ranged from 0.03 to 0.31. The most diverse was number of spikelets per panicle and it varied between 16.1 for PL 51742_(39)_ and 87.9 for PL 51199_(8)_. A ratio of glumes length (1.04–1.19) and a ratio of lower glume length to spikelet length (0.88–0.99) were almost uniform within the set of accessions. The range of qualitative traits diversity was similar to the quantitative ones. The highest variation was observed for rigidity of flag leaf (0.32) while the lowest was for leaf-blades twist (0.05).

**Table 2:** The list of morphological traits. Each trait with the scale or units with variation coefficient for quantitative traits or the unbiased genetic diversity coefficient for qualitative traits

No.	Trait	Measure	Variation^a^
1	1000 grains weight	g	0.16
2	Awn insertion	mm	0.08
3	Days to heading	Day	0.09
4	Length of awn	mm	0.17
5	Length of flag leaf	cm	0.18
6	Length of lemma	mm	0.08
7	Length of lemma tip	mm	0.20
8	Length of lower glume	mm	0.08
9	Length of spikelets	mm	0.09
10	Length of rachilla	mm	0.08
11	Length of upper glume	mm	0.08
12	Length of upper internode	cm	0.13
13	Number of nodes in panicle	No.	0.14
14	Number of spikelets per panicle	No.	0.31
15	Number of tillers	No.	0.27
16	Number of veins in lower glume	No.	0.07
17	Plants height	cm	0.16
18	Position of awn insertion	mm	0.11
19	Ratio of length of glumes	ratio	0.03
20	Ratio of length of lemma to length of lemma tip	ratio	0.17
21	Ratio of length of lower glume to length of spikelet	ratio	0.03
22	Width of flag leaf	mm	0.21
23	Angle of flag leaf to culm	Acute; Intermediate; Obtuse	0.30
24	Angle of second leaf to culm	Acute; Intermediate; Obtuse	0.24
25	Erectness of spikelets	Erect; Drooping	0.24
26	Hairiness of leaf blade	Glabrous; Pubescent	0.10
27	Hairiness of leaf sheath	Glabrous; Pubescent; Highly pubescent	0.13
28	Hairiness of lemma of lower flower	Glabrous; Slightly pubescent; Pubescent	0.13
29	Hairiness of lemma of upper flower	Glabrous; Slightly pubescent; Pubescent	0.07
30	Hairiness of rachilla	Glabrous; Single hairs at upper end; Hairs at upper end; Pubescent	0.29
31	Intensity of flag leaf spirality	Lack of rotation; week rotation (1/4 torsion); medium rotation (1/2 torsion); strong rotation (3/4 torsion); very strong rotation (1 torsion)	0.29
32	Leaf-blades twist	Anticlockwise; Clockwise	0.05
33	Lemma colour	White; Light-grey; Dark-grey; Black	0.18
34	Lemma tip type	Biaristulate; Bisetulate-biaristulate	0.24
35	Rigidity of flag leaf	Stiff; Deflected; Bent; Strongly bent	0.32
36	Rigidity of second leaf	Stiff; Deflected; Bent; Strongly bent	0.19

^a^Variation – the variation coefficient for quantitative traits (1–22) or the unbiased genetic diversity coefficient for qualitative traits (23–36)

**Fig. 1:**
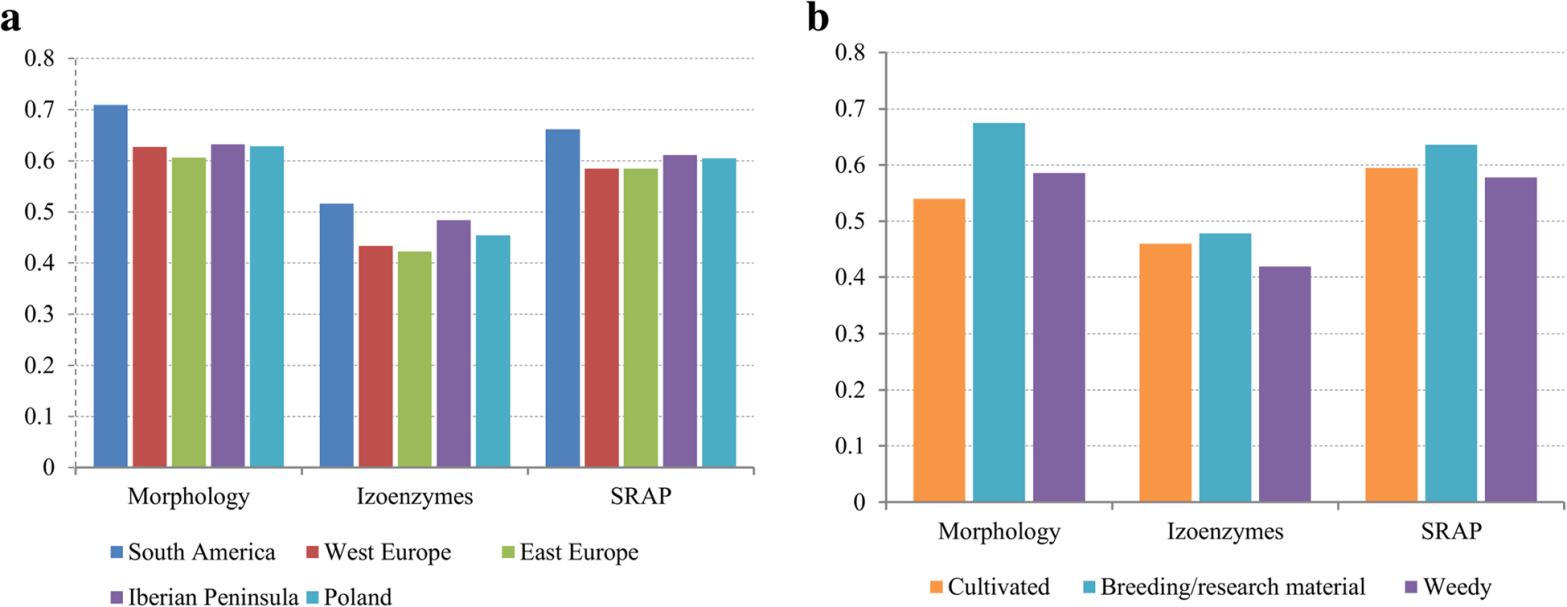
The results of Shannon-Weaver (H′) coefficient. Calculation performed within geographical regions and groups based on improvement status of sand oat accessions

The diversity index of the morphological traits was calculated for groups based on the major geographical regions i.e. West Europe, East Europe and South America. Additionally, two minor groups were separated i.e. the Iberian Peninsula and Poland (Fig. 1a). The most variable were accessions from South America (H′ = 0.709) while the East European ones were the least diverse (H′ = 0.607). The extraction of accessions originated from the Iberian Peninsula demonstrated that their differentiation is higher (H′ = 0.632) than in the whole West Europe. Also, Polish accessions were a bit more diverse (H′ = 0.629) than the European ones. The classification by the improvement status indicated the highest morphological variation within the breeding/research materials (H′ = 0.675) while the lowest was in the group of cultivated accessions (H′ = 0.54) (Fig. 1b).

**Fig. 2:**
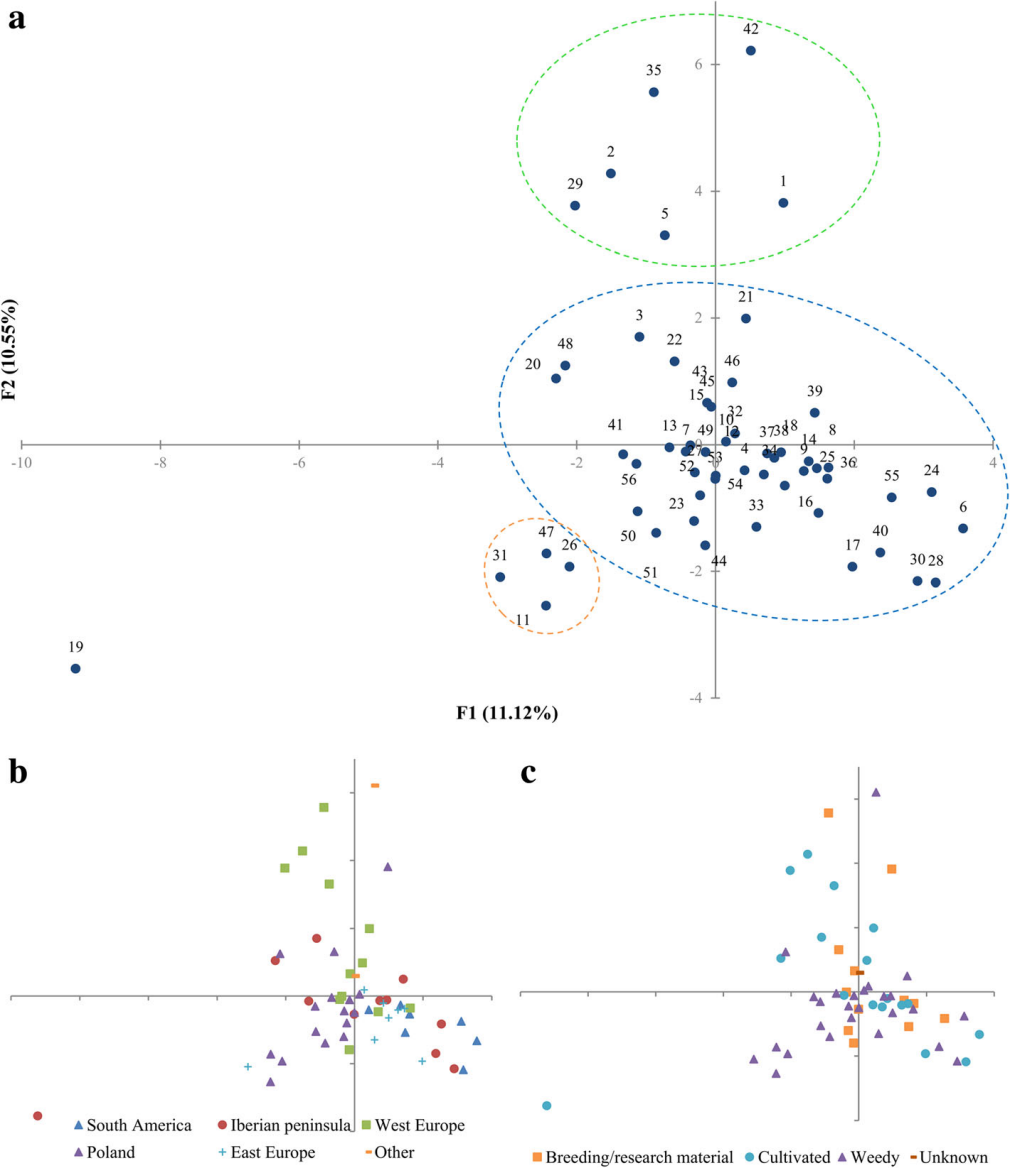
The scatter plots of MFA results. **a** the plot with order numbers according to Table 1; **b** the plot with indication of geographic regions; **c** the plot with improvement status

The results of multiple factor analysis revealed that only 31% of variability was explained by the first three factors. In the scatter plot of the first two factors sand oat accessions formed three groups. The biggest was composed of 45 accessions and was in the centre of coordinate system. Two smaller groups were also identified, and they were formed by four and six accessions respectively. The accession PL 51582_(19)_ originated from Spain was distinctive from the above-mentioned groups (Fig. 2a). The detailed analysis of the plot according to the geographic origin of accessions revealed some additional differences. Seven accessions originated from South America exhibited phenotypic similarity to accessions from the Iberian Peninsula and distinctiveness from accessions acquired from Poland (Fig. 2b). Classification by the improvement status indicated that majority of weedy accessions diversity was reflected in cultivated forms or breeding/research materials (Fig. 2c).

### Isoenzymes

Seven, out of 12 tested, isoenzymatic systems were polymorphic (Table 3), and they allowed to analyse 14 loci. Five isoenzymatic assays allowed to analyse more than one locus ie. two in peroxidase (PRX), diaphorase (DIA) and malate dehydrogenase (MDH) and three in acid phosphatase (ACP) and aspartate aminotransferase (AAT). Unfavourably, most of them were linked or uniform thus they significantly reduced the informativness. Ten out of 26 alleles had frequency below 0.05 so they were identified as unique. The “null” alleles were detected in the PRX and glucose-6-phosphate isomerase (GPI) assays. The mean value of Polymorphic Information Content was rather low (0.21) and it was in the 0.04–0.52 range for the AAT/DIA and ACP, respectively.

**Table 3:** The list of analysed enzymatic systems

No.	Enzyme	Abbreviation	EC number	PIC
1	Leucine aminopeptidase	LAP	EC 3.4.11.1	0
2	6-Phosphogluconate dehydrogenase	6PGD	EC 1.1.1.44	0
3	Diaphorase	DIA	EC 1.6.99.	0.04
4	Shikimate dehydrogenase	SKDH	EC 1.1.1.25	0.07
5	Aldolase A	ALDO	EC 4.1.2.13	0
6	Isocitrate dehydrogenase	IDH	EC 1.1.1.42	0
7	Acid phosphatase	ACP	EC 3.1.3.2	0.52
8	Malate dehydrogenase	MDH	EC 1.1.1.37	0.47
9	Phosphoglucomutase	PGM	EC 5.4.2.2	0
10	Aspartate aminotransferase	AAT	EC 2.6.1.1	0.04
11	Peroxidase	PRX	EC 1.11.1.7	0.14
12	Glucose-6-phosphate isomerase	GPI	EC 5.3.1.9	0.17

*EC number* Enzyme Commission number, *PIC* Polymorphic Information Content coefficient

In the set of 56 sand oat accessions 17 different isoenzymatic profiles were detected. Forty-three accessions belong to four major profiles represented by 17, 11, 8 and 7 accessions respectively. Thirteen profiles were unique i.e. were identified only in one accession in the set. Among Spanish accessions four profiles were detected including the unique one. The accessions originated from Portugal represented three profiles and one was also unique. Two profiles were found in French accessions, but the difference was found only in MDH. Five unique profiles were identified among eight profiles of Polish accessions. For more details see Table 1.

The Shannon-Weaver index of isoenzymatic data was lower than in morphology but configuration of groups remained unchanged (Fig. 1a, b). The analysis of molecular variance (AMOVA) indicated that the majority of isoenzymatic variation occurred within the countries (84%) whereas there was no variation among countries and the remaining 16% was detected among three geographic regions. Only 4% of variance occurred among groups with different improvement status.

**Fig. 3:**
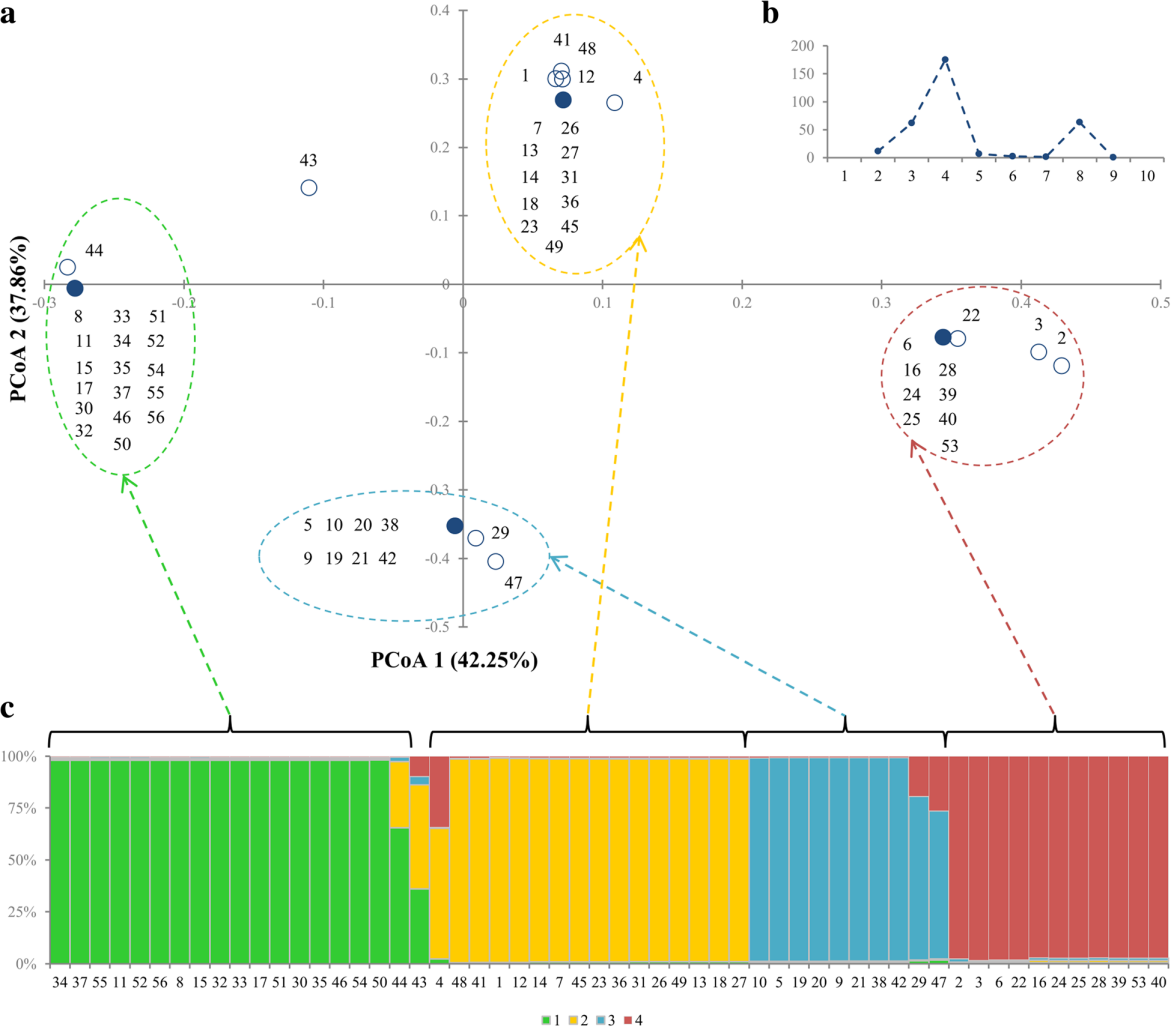
The results of isoenzymatic analysis of 56 accessions of sand oat. **a** the plot of PCoA with order number corresponding to Table 1. The black points indicated multiple accession match; **b** the results of ad hoc measure ∆K (72) generated by CLUMPAK software.; **c** the results of 100,000 iterations of STRUCTURE software with K values k = 4 where K is the number of populations assumed; each vertical bar represents one accession that is marked by borders and order number (Table 1). The length of the coloured segment shows the estimated proportion of membership of that sample to each group. The arrows indicate the consistent results of PCoA and Bayesian analysis

Principal Coordinate Analysis revealed that 86.12% of variability was explained by the first three coordinates. The scatter plot of the first two coordinates confirmed the earlier observation of limited informativeness of isoenzymatic assays (Fig. 3a). Most of the accessions were concentrated in four distinctive major points. Single differences making some profiles unique were reflected as small displacements of the accessions outside the major points. In fact, only accession PL 51746_(43)_, that origin remains unknown, as the only one did not match to any of four groups.

STRUCTURE analysis revealed the peak distribution of ΔK occurred at K = 4 for 10 simulations at K values from 1 to 10 (Fig. 3b). This indicated the presence of four clusters (Fig. 3c) that were consistent with PCoA results. Out of 56 accessions, only three (PL 51022_(4)_; PL 51746_(43)_ and PL 51747_(44)_) could not be assigned to clusters based on 70% membership threshold, meaning that they were considered to have admixed parentage (Fig. 3c). Neither PCoA nor STRUCTURE analysis showed compatibility with the geographic origin or the improvement status. A detailed analysis of the composition of each cluster revealed that accessions containing grains with black lemma and also with low thousand grains weight were grouped in the fourth cluster. Whereas accessions with thousand grains weight far above average, short and narrow flag leaves, long rachillas, high insertion of awns and high ratio of lemma length to lemma tip length were placed in the first cluster.

### SRAP

Distinct DNA profiles were obtained for all 56 accessions by eight SRAP primers pairs. Fragments sizes ranged from 50 bp to 828 bp. A total of 589 fragments were amplified, 53% of them were polymorphic (*p* < 0.95). As many as 324 fragments appeared with very low frequency (*p* < 0.05) and were treated as unique. Finally, only 16 fragments were monomorphic in tested set of sand oat accessions. The PIC was in the 0.26–0.41 range (Me3/Em4 and Me4/Em7 respectively) with mean 0.33 (Table 4).

**Table 4:** The list of SRAP primers combination used in the study

No.	Primers pair	Me primer sequence	Em primer sequence	NF	%PF	PIC
1	Me1/Em3	TGA GTC CAA ACC GGA TA	GAC TGC GTA CGA ATT GAC	92	55.40%	0.31
2	Me1/Em4		GAC TGC GTA CGA ATT TGA	84	45.20%	0.33
3	Me2/Em8	TGA GTC CAA ACC GGA GC	GAC TGC GTA CGA ATT CAC	90	63.30%	0.3
4	Me2/Em10		GAC TGC GTA CGA ATT CAT	59	66.10%	0.39
5	Me3/Em4	TGA GTC CAA ACC GGA AT	GAC TGC GTA CGA ATT TGA	91	44.00%	0.26
6	Me3/Em7		GAC TGC GTA CGA ATT CAA	68	30.90%	0.3
7	Me4/Em6	TGA GTC CAA ACC GGA CC	GAC TGC GTA CGA ATT GCA	60	58.30%	0.31
8	Me4/Em7		GAC TGC GTA CGA ATT CAA	45	71.10%	0.41

*NF* Number of fragments, *%PF* Percent of polymorphic fragments (*p* = 0.95), *PIC* Polymorphic Information Content]

The Shannon-Weaver index of genetic data was calculated for the same groups as for morphology and isoenzymes (Fig. 1). In general H′ value was higher than for isoenzymes but lower than for morphology. The overall pattern of diversity within groups remained the same as in the above-presented analysis. AMOVA did not show any variation among three geographic regions. Only 3 % of variation was present among countries and the rest occurred within countries. The groups with different improvement status differed from each other only 3% of the molecular variance.

**Fig. 4:**
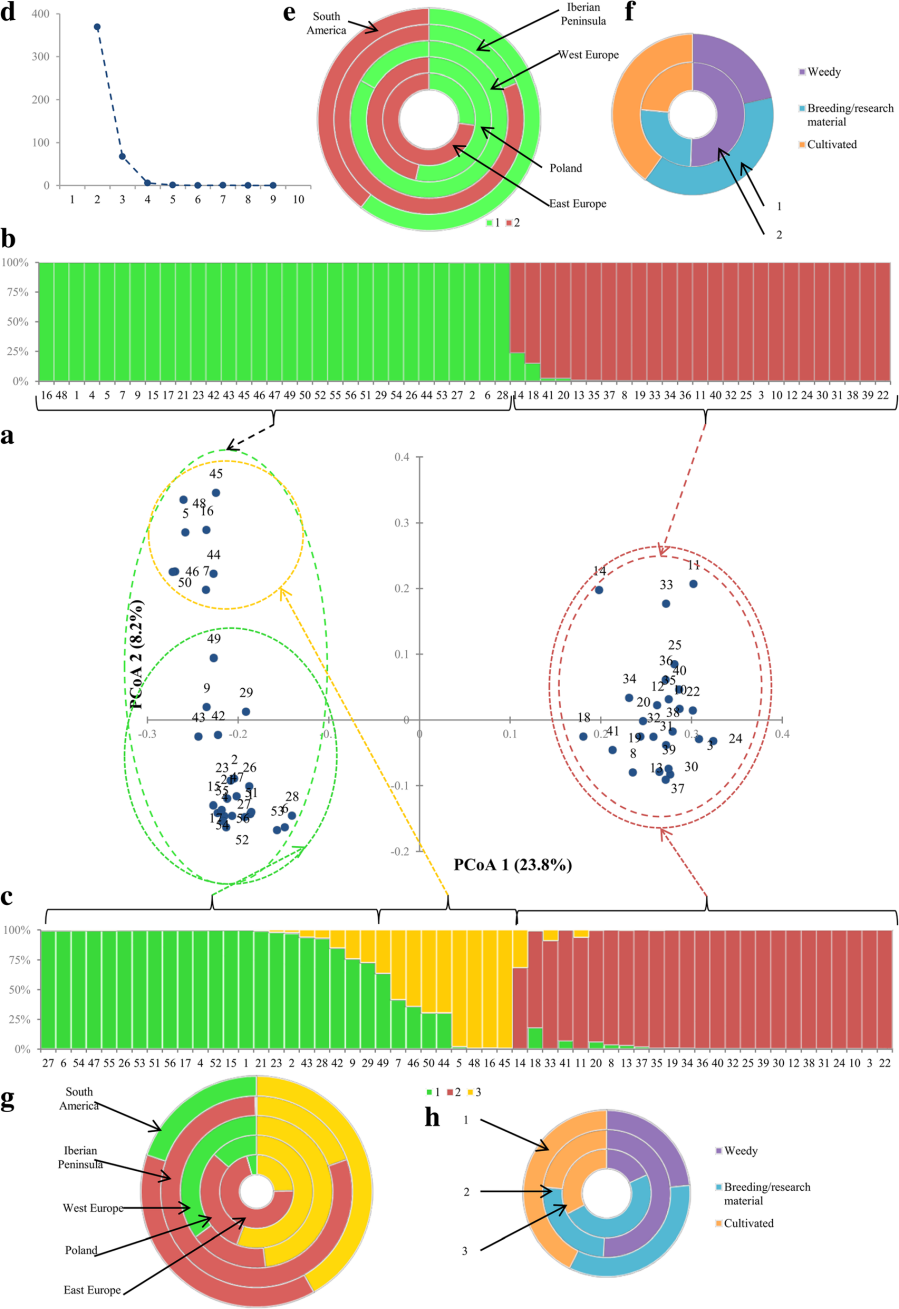
The results of genetic analysis based on SRAP markers. **a** the plot of PCoA results with order number corresponding to Table 1; **b** the results of 100,000 iterations of STRUCTURE software with K values k = 2 where K is the number of groups assumed; each vertical bar represents one accession that is marked by borders and order number (Table 1). The length of the coloured segment shows the estimated proportion of membership of that sample to each group. The arrows indicate the consistent results of PCoA and Bayesian analysis; **c** the results Bayesian analysis where values k = 3; **d** the results of ∆K measure (72) obtained from CLUMPAK software.; **e** the ring chart presents the participation of two STRUCTURE groups in geographic regions (numeration in accordance with b diagram); **f** the ring chart presents the participation of two STRUCTURE clusters in accessions with different improvement status (numeration in accordance with b diagram); **g** the ring chart presents the participation of three STRUCTURE groups in geographic regions (numeration in accordance with c diagram); **h** the ring chart indicates the participation of three STRUCTURE clusters in accessions with different improvement status (numeration in accordance with c diagram)

PCoA analysis was performed using Dice distance matrix to graphically summarize the genetic diversity among 56 sand oat accessions. The first three coordinates accounted for 36.1% of total variance. PCoA plot was made using first two coordinates (Fig. 4a). The accessions were distributed into two separate groups composed of 31 and 25 ones. All Spanish accessions were placed in the second group. Only two accessions from eastern Europe (PL 51499_(9)_ and PL 51579_(17)_) were assigned to the first group. South American accessions were distributed into both groups as follows: the Brazilian sand oats were in the first group, the Chilean ones were placed in the second, while the Uruguayan accessions were split in two groups. Polish accessions also were scattered. Three French accessions were placed in the first group, but PL 51584_(21)_ was distinctive from the other two.

Bayesian analysis was conducted to determine the genetic structure among sand oat accessions (Fig. 4b,c). The maximum ΔK occurred at k = 2 and in the further order but much lower for k = 3 (Fig. 4d). Considering k = 2, the set was split into two sub-groups (group 1, group 2) containing 31 and 25 accessions. All of accessions were assigned to the groups based on 70% membership threshold i.e. no admixture was observed. Based on bar plot (Fig. 4b) and ring chart (Fig. 4e) it can be assumed that accessions from South America were in both groups. Most of the accessions originated from the Iberian Peninsula was placed in the second group but two Portuguese (PL 51757_(53)_ and PL 51759_(55)_) belonged to the first one. Sand oats from West Europe were in the first cluster except two from Germany (PL 51738_(35)_ and PL 51739_(36)_) whereas the East European ones were placed in the second group except PL 51499_(9)_ and PL 51579_(17)_. Polish accessions were evenly distributed in both groups. All of the above exceptions to the general grouping pattern applied to materials influenced by breeding.

Group one consisted mainly of improved materials but also included ten weedy accessions from Poland and Ethiopia (Fig. 4f). Conversely the second group was composed of weedy accessions supplemented by three breeding/research materials from Poland and Germany and four cultivated accessions from Spain, Portugal and Bulgaria. An internal structure occurrence was indicated by the lower value of ∆k for k = 3 (Fig. 4c). The third group was separated from the first one. Four accessions were classified in it (PL 51105_(5)_, PL 51578_(16)_, PL 51748_(45)_ and PL 51752_(48)_) and some others were predicted to have their origin from at least two sources of diversity. The presence of the third group perfectly reflect botanical diversity of three French accessions i.e. PL 51584_(21)_ classified as var. *strigosa* placed in the first group, PL 51105_(5)_ a mixture of var. *strigosa* and *intermedia* was assigned into third group, while PL 51749_(46)_ the most botanically diverse (var. *strigosa, intermedia* and *gilva*) had certain level of admixture between the group one (0.336) and three (0.643). Within the groups of accessions originated from Iberian Peninsula and East Europe the level of admixture was the lowest, whereas the highest level was observed in the West European and South American group (Fig. 4g). The admixture level of Polish accessions corresponded to the geographic location i.e. it was between the levels of West and East European groups. Overall, the results from Bayesian approach implemented in STRUCTURE and PCoA were consistent.

### GPA

**Fig. 5:**
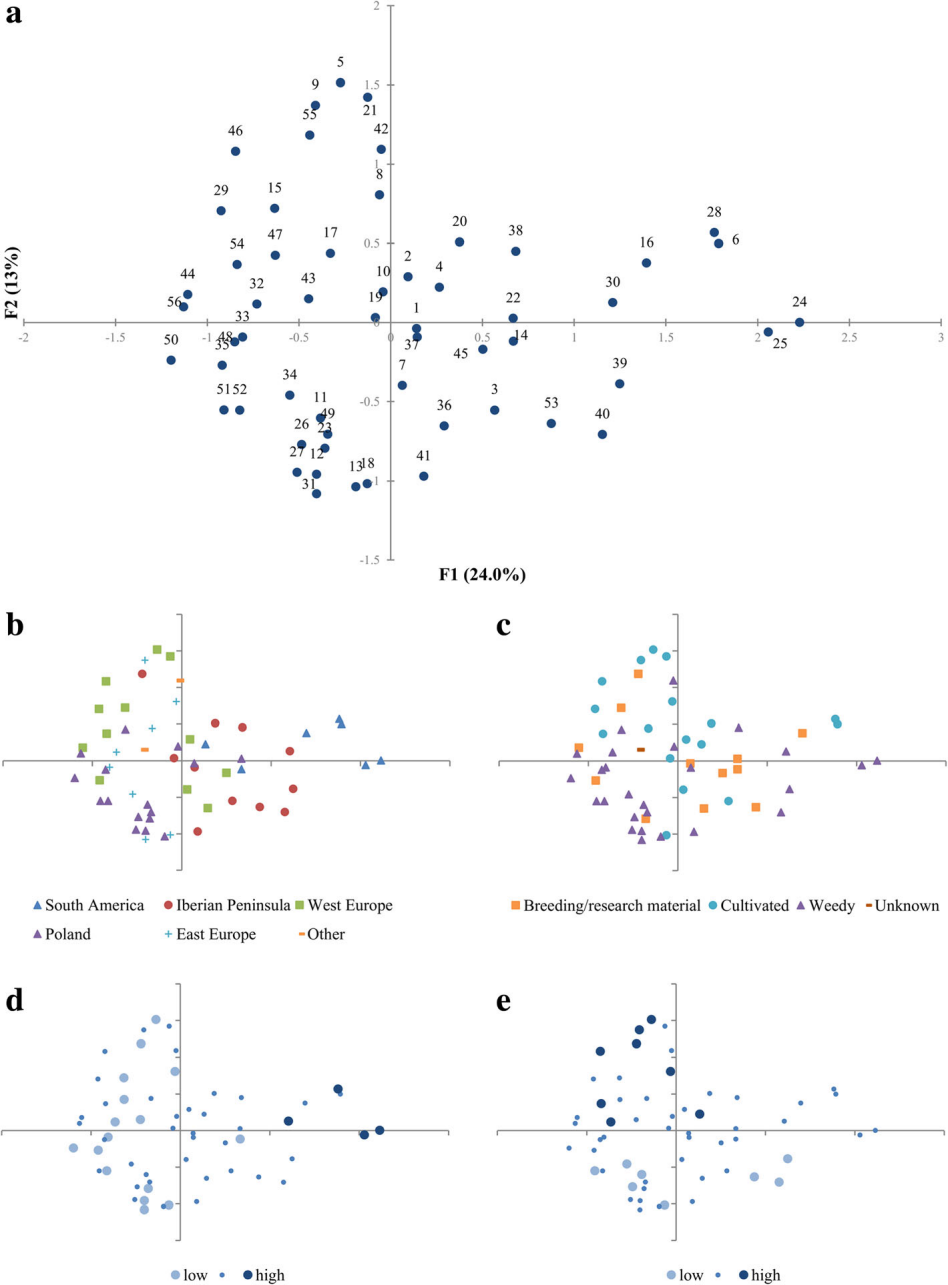
The results of GPA analysis. **a** the general plot with numbers corresponding to Table 1; **b** the plot with indication of geographic regions; **c** the plot indicates the improvement status; **d** the plot indicates a 1000 grain weight; **e** the plot indicates a plant height

The consensus configuration based on morphological, isoenzymatic and genetic data was obtained by Generalized Procrustes Analysis. The first three coordinates were responsible for 24, 15.4 and 13% (∑ = 52.4%) of total variance respectively. The first coordinate was strongly negatively correlated with both latitude and longitude. The projection of accessions on bi-plot of Coord. 1 and Coord. 2 (Fig. 5) revealed that the most of South American materials were located rightmost (Coord.1 > 1.4). The accessions originated from the Iberian Peninsula were placed in the 0.1–1.25 range of Coord.1, while the rest of European accessions took values below zero of Coord. 1. Correlations with quantitative morphological traits were also considered. Coord.1 was positively correlated with thousand grain weight and Coord.2 was also positively correlated with plant height (Fig. 5d, e). Several other traits were significantly correlated with the first two GPA coordinates and detailed results were shown in Table 5.

**Table 5:** The results of correlation analysis of GPA coordinates with geographical data and morphological traits

	Coord. 1	Coord. 2
Longitude	−0.734	*ns*
Latitude	−0.751	*ns*
1000 grains weight	0.688	*ns*
Awn insertion	0.619	*ns*
Days to heading	*ns*	−0.310
Length of awn	−0.653	*ns*
Length of flag leaf	− 0.580	*ns*
Length of lemma	−0.289	− 0.424
Length of lemma tip	−0.536	− 0.310
Length of lower glume	−0.335	− 0.478
Length of spikelets	−0.432	− 0.440
Length of rachilla	0.387	*ns*
Length of upper glume	*ns*	−0.500
Length of upper internode	*ns*	0.561
Number of nodes in panicle	−0.514	0.477
Number of spikelets per panicle	−0.378	0.566
Number of tillers	*ns*	*ns*
Number of veins in lower glume	0.605	*ns*
Plants height	*ns*	0.696
Position of awn insertion	0.717	0.328
Ratio of length of glumes	−0.366	*ns*
Ratio of length of lemma to length of lemma tip	−0.499	*ns*
Ratio of length of lower glume to length of spikelet	0.487	*ns*
Width of flag leaf	−0.638	*ns*

## Discussion

The use of genetic resources depends on the access to information from evaluation and characterization of collected accessions, and only the combination of data from many different types of experiments leads to complete information [25, 26]. Management of genetic resource data is one of the main gene banks activities, but its quality is highly variable. These problems result from the maladjustment of existing databases (software and hardware) to handle such data. Historical data from previous analyses are also very rarely used in development of results for genetic resources characterisation and integrated data studies from many different analyses are even less frequent. In this context, presented studies on the Polish NCPGR collection seem particularly important. Historical morphological data are useful for research, breeding and genetic resources characterisation. Such data are suitable for genomic selection as they usually offer higher phenotypic accuracy due to replicated trials in different years and/or locations [27]. Historical data were used e.g. for association mapping in wheat and barley and for genomic selection in wheat [27–30]. In the presented paper, data were originated in 1980’s so quantitative traits values may differ from contemporary results especially in the context of climate change. The interaction between genotype and environment is an important factor for breeders and agronomists as its high level can significantly prolong the breeding process. The qualitative traits on which the botanical description was based are considered to be stable and highly inherited [31]. The morphological description was made regarding the initial characteristics of the collection’s variability and not to the direct use of the results for breeding programs or for the estimation of its utility value. For these purposes, the collection should be subjected to further multiplied field trials.

### Morphology

In this paper the data from the description of plant phenotype (including botanical variety) were analysed. Precise botanical identification is scarce in the germplasm databases as it requires from the curator to have expertise in botany. Tis kind of information, especially in case of self-pollinating plant species, can be the first indicator of internal accession diversity and can be used as a genetic integrity marker. Botanical differentiation at sub-species level has been identified earlier, for example for *Avena sativa* L., *A. strigosa* and *Triticum monococcum* L. [14, 32, 33]. Among the 56 accessions investigated here, only seven out of 17 botanical varieties were found. The most numerous varieties were *strigosa* and *gilva*, whose kernels have grey or brown lemma respectively. Presence of genes for the other colours is not excluded, if to consider inheritance mode of the colour of the lemma. In oats five colours of lemma were identified, i.e. black (including dark brown), grey, red, yellow and white which are fairly stable [34]. It is postulated that black lemma colour is epistatic over the other four while grey is hypostatic to black, epi- or hypostatic over red and epistatic over yellow and white [35]. The absence of remaining botanical varieties in the examined materials is presumably a consequence of the limited sample size. However, in order to determine whether the botanical variability of the species has been preserved, it is recommended to verify in this matter all collected accessions starting from those originated from the Iberian Peninsula, i.e. the place where the sand oat evolved [36].

South American accessions had a high 1000 grains weight, which may result from the use of *A. strigosa* in that region mainly as forage crop and, therefore, not paying special attention to grain yields [1]. There are reports about negative correlation between the weight of 1000 grains and yield in common oat [37, 38]. These accessions also had short awns, which is also due to the way they were used, since long awns cause injuries and the ensuing gingivitis and stomatitis in horses [39, 40]. Morphological characteristics of the breeding/research materials may also be related to breeding process towards improving yield and quality of grain, as indicated by an increase in the number of spikelets and a reduction in the number of rachillas and glumes. Morphological features of the accessions collected in Poland reflect their weedy nature. The lower similarity between West European accessions compared to those from East Europe is due to the presence of a larger number of biotypes in that region. Populations distant from species diversity centre have a reduced number of biotypes and this phenomenon is described as biotypes depletion [41]. The pool of morphological traits presents in breeded and weedy forms is similar that indicates a short-term and extensive breeding process, however, all accessions, which we can classify as cultivated, were characterized by better grain and green mass parameters in comparison with the weedy ones. In order to select accessions useful for breeding and/or direct field cultivation as an alternative crop, it is necessary to perform a more detailed assessment of yield-forming and especially grain qualitative traits. However, based on the results presented in this paper, it can be assumed that at least accessions PL 51752_(48)_ and PL 51575_(15)_ may be valuable genetic stocks for these purposes.

### Biochemistry

Since their discovery by Hunter and Markert in 1957, isoenzymes have played a key role in many fields of biology over several decades [42]. However, the development and spread of techniques using the nucleic acid polymorphism gradually limited their importance, finally leading to their complete marginalisation. However, the time in which they were a leading research tool resulted in numerous studies on the characteristics of genetic resources [43–47]. The results of these studies are nowadays rarely used and compared with current research results, even if it concerned the same set of accessions. In this paper results of isoenzymatic analyses enriched genetic variability analysis of *A. strigosa* based on SRAP molecular markers and morphology. For the analysis of 56 accessions preserved in the Polish collection of *Avena*, 12 enzymatic systems were used, which had been previously analysed and described by other authors [43, 48]. Seven of them detected polymorphism in the investigated accessions set (AAT, ACP, DIA, GPI, MDH, PRX, and SKDH), while as many as 10 alleles had a very low frequency, i.e. below 0.05. In our earlier study, only two (ACP and MDH) out of the same 12 systems, showed the presence of varied alleles in eight landraces of sand oat [14]. In Kubiak’s paper [8], in which 19 *A. strigosa* ecotypes were analysed, only two (MDH and EST - esterases) out of six applied systems showed polymorphism. However, research on 1005 accession of *A. sterilis* showed that out of 134 alleles, obtained by the use of 23 isoenzymatic systems, only 10 were not polymorphic and 54 alleles occurred in less than 10% of accessions [43]. These markers also showed relationships with geographical origin, which was not possible to detect by SRAP markers in the course of our study. Based on the above-mentioned results, it can be concluded that the usability/resolution of isoenzymes depends on the number of studied accessions and their geographical origin. The obtained results showed dissimilarity of morphological and biochemical results, that is a phenomenon commonly described in literature [49–51].

### Genetics

In this study eight combinations of SRAP starter pairs were used, which produced a total of 589 fragments, 53% of which were polymorphic, and this value was significantly lower than indicated by the available literature data for other species. In the study of 16 Iraqi wheat cultivars using 28 primer pairs, over 87% of the fragments were polymorphic [52]. 95% in the study of, 96% of polymorphic loci were also analysed In *Cynodon dactylon* (L.) Pers., and 53 *Buchloe dactyloides* genotypes polymorphism was found at the level over 90% [53, 54]. In the phylogenetic study of *Festuca-Lolium* complex all obtained fragments were polymorphous [55]. A lower detected level of polymorphism may result from the analysis of pooled samples from accessions with a high level of internal variability [14]. However, it may also result from the flowering biology, i.e. *A. strigosa* is a self-pollinating species or it may prove that the genetic pool of this species is quite narrow. The values of PIC coefficient were consistent with literature data, however, they were lower than those obtained by using ISSR markers for eight sand oat accessions [14]. This is probably due to differences in the type of analysed genome regions i.e. SRAPs amplify coding regions while ISSRs amplify both coding and non-coding regions. Nevertheless, the sensitivity of the method was sufficient to distinguish unequivocally all the accessions tested.

The results of PCoA and model-based clustering agreed with existence of two major clusters, as well as presence of a secondary structure i.e. two sub-groups in the largest group. The general pattern of clustering is that the accessions from the Iberian Peninsula were placed in the second cluster except for two Portuguese ones (PL 51757 and PL 51759). These two accessions were classified as a breeding/research material thus they genetic makeup might be distinct from the native gene pool. It is also worth to consider whether a small sub-group, marked in the diagram as No 3, may reflect a gene pool characteristic for Great Britain. In that region *A. strigosa* has a long tradition of cultivation and was the main oat species till seventeenth century [56]. However, to confirm this assumption, it would be necessary to examine more accessions from that region.

South American accessions showed the highest value of diversity coefficient and were assigned to three different groups. It seems to confirm the hypothesis about secondary diversity of sand oat in South America [57]. Detailed analysis of population structure led to another essential finding that the effect of anthropogenic factor is noticeable. Weedy-status accessions accounted for more than half of Group 2, less than a quarter of Group 1 and roughly a fifth part of Group 3. The lack of distinctiveness of groups with different levels of improvement was linked to extensive type of breeding and its relatively short duration.

### Joint analysis

The majority of studies on the characterisation of genetic resources do not use the available statistical tools to integrate data from different types of analysis, i.e. phenotypic and genetic data. A joint analysis is essential in order to obtain a more reliable, complete description of genetic resources, which should result in their better utilization.

Generalised Procrustes Analysis (GPA) allows using the most appropriate ordination method for each type of data i.e. MFA in phenotypic data and PCoA in biochemical and genetic data. The final configuration is the average of all data after their initial transformation. Based on these results, it can be concluded that a secondary centre of diversity is being created in South America and that it has its genealogy from the Iberian Peninsula. Materials from the Iberian Peninsula link the gene pool characteristic for South America and the one present in other regions of Europe. It is clear that the selection in these two regions took place independently and in different directions and its source should be considered both an anthropogenic and an environmental factor.

### Genetic integrity and duplicates

Based on passport data, two groups of accessions were selected, which potentially represent a multiplication of one original sample. This applies to three accessions from France (PL 51105_(5)_, PL 51584_(21)_ and PL 51749_(46)_) and three from Brazil (PL 51022_(4)_, PL 51149_(6)_ and PL 51730_(28)_). French accessions originate from a sample collected by N.I. Vavilov himself that is stored by N.I. Vavilov Research Institute of Plant Industry in St. Petersburg under the accession number VIR 2172. Each of them was obtained from a different donor institution i.e. PL 51105_(5)_ was obtained from Genbank Department, Division of Genetics and Plant Breeding, Research Institute of Crop Production, Prague, Czech Republic; PL 51584_(21)_ from Plant Genetics and Germplasm Institute, Beltsville, USA and PL 51749_(46)_ from Institute of Plant Genetics and Crop Plant Research, Gatersleben, Germany. In each case, seeds, replicated outside the primary institution, were delivered to Poland. Differences between the three accessions were visible at each stages of the analysis, i.e. starting from the botanical composition and ending with genetics. The results showed that we are dealing with a loss of genetic integrity which is resulting from a genetic drift caused by the multiplication of an insufficient number of seeds provided by the donor institution to create a duplicate in another facility. A comparison of internal variation of these accessions with their analogues in three gene banks, and reference of all of them to the original accession would reveal exactly what processes caused the differentiation of these samples and when the major changes were introduced. Taking into account all obtained results, it should be excluded that at any time contamination with foreign seeds has occurred. In the present situation it is necessary to consider whether it is more sensible to maintain the *status quo* or to combine them into one accession which will better reflect the initial variability.

Three Brazilian accessions have the same cultivar name in the passports i.e. Saia. This is an old Brazilian cultivar introduced to the State of Rio Grande do Sul in the early 1940’s, bred through selection from local population [58, 59]. According to passport data, all these accessions were introduced to the gene bank collection through Polish breeding stations in Borowo (PL 51022_(4)_), and Wielopole (PL 51149_(6)_ and PL 51730_(28)_). Only in case of one of them we have an accession number from a gene bank in the USA. The origin of remaining two is unknown. All three accessions have the same botanical composition i.e. are a mixture of var. *strigosa* and *gilva*. However, the results of all analyses indicate similarity between two accessions received from the station in Wielopole and distinctiveness of the accession from Borowo. In this case we can state that accessions PL 51149_(6)_ and PL 51730_(28)_ are duplicates which arisen as a result of double materials submission to the gene bank in a few years interval both originate from CIav 4639 preserved by USDA-ARS National Small Grains Collection, Aberdeen, USA. Due to the lack of data on the origin of the third sample and its certain distinctiveness manifested at all levels of the performed analysis, it is impossible to state unequivocally whether it is a duplicate that has lost its genetic integrity, or whether it is a distinct accession.

### To be or not to be a successful crop

To be successful as a crop in today’s commercialised world it is essential that *A. strigosa* should be subjected to a sustainable breeding process. Due to low yield, i.e. two times lower than in common oat and significantly smaller seeds, currently this species has little chance of being returned to cultivation. It seems reasonable to promote its usefulness for cultivation in extensive organic farms to produce functional food. Sand oat has many features that are seen as valuable in this type of farming. Based on unpublished studies (Podyma unpublished), it can be concluded that it is suitable for cultivation on very weak, acidic soils and in mountain conditions. It tolerates weed infestation and agrotechnical deficiencies much better than *A. sativa*. It can be cultivated without chemical protection against fungal diseases such as powdery mildew or crown rust. Sand oat can be an important complementary grain crop because it has a higher protein content (16.0% air-dry matter) than oats (10.5%). A higher content of beta-glucan and polyphenols in dehusked grains in comparison to common oat was found, that proves high pro-health value of the product. This species does not seem to have the potential to replace commonly cultivated cereals such as wheat, barley or even rye. A balanced breeding programme that would improve the profitability of the crop but would not significantly reduce genetic variability would be advisable.

The information on comprehensive, multi-level research on sand oat is missing. Despite the relatively large representation of this species in various gene banks, it is highly probable that the vast majority of stored worldwide accessions are duplicates, and the protected gene pool is relatively narrow. Considering that this species is not known to occur in the wild-state and its spread and survival is inextricably linked to humankind, the verification of existing genetic resources as well as the acquisition of new samples from areas where it is still cultivated, conserved on farms or occurs in the common oat fields is a necessary activity for the sustainable use of this species in agriculture. Further research on sand oat, carried out by our team, focuses on the evaluation of agronomic and quality characteristics in organic farming. We hope that in the near future we will be able to promote sand oat for a new, healthy, trendy, alternative crop.

## Conclusions

- Majority of weedy accessions diversity was reflected in cultivated forms or breeding materials and it is a derivative of relatively brief and extensive breeding of *A. strigosa*.
- The second centre of *A. strigosa* diversity is being created in South America and it originates from populations from the Iberian Peninsula.
- *A. strigosa* meets all the requirements for alternative crop species, but further studies are needed to identify the genotypes/populations with the most favourable distribution of utility and quality parameters

## Methods

### Plant material

Fifty-six sand oat accessions representing diverse origin were obtained from long term storage of National Centre for Plant Genetic Resources, Radzików, Poland (Table 1). The accessions derived from 15 different countries and in the case of one the origin has remained unknown. Seven accessions originated from South America, one from Africa while the rest came from European countries among which the most numerous were Polish. The accessions were collected between 1917 and 1990 and the oldest ones were collected by N.I. Vavilov during his expeditions.

### Botanical identification

Botanical varieties were identified based on intra-specific taxonomic systems of genus *Avena* L. according to Rodionova et al. [60]. The classification is based on clearly recognisable morphological traits such as shape of panicle, colour and pubescence of lemma, length of glumes, awnedness, character of disarticulation of florets in a spikelet and characteristics of the caryopsis. An overview of *A. strigosa* botanical varieties is included in Table 6. The evaluation was carried out under laboratory conditions for 10 plants representing the variability of accession.

**Table 6:** Description of botanical varieties based on Rodionova et al. [60]

	Botanical variety	Description
1	*albida* Marq.	Equilateral panicle; Glabrous and white lemma
2	*strigosa* Rod et Sold.	Equilateral panicle; Glabrous and grey lemma
3	*gilva* Mordv.	Equilateral panicle; Glabrous, and brown lemma
4	*melanocarpa* Mordv.	Equilateral panicle; Glabrous and black lemma
5	*flava* (Marq.) Mordv.	Equilateral panicle; Hairiness of awn insertion; Yellow lemma
6	*intermedia* Marq.	Equilateral panicle; Hairiness of awn insertion; Grey lemma
7	*nigra* Marq.	Equilateral panicle; Hairiness of awn insertion; Black lemma
8	*alba* Marq.	Equilateral panicle; Pubescent and white lemma
9	*fusca* Marq.	Equilateral panicle; Pubescent and grey-brown lemma
10	*candida* Mordv.	Flagged panicle; Glabrous and white lemma
11	*tephera* Mordv.	Flagged panicle; Glabrous and grey lemma
12	*hepatica* Mordv.	Flagged panicle; Glabrous and brown lemma
13	*nigricans* Mordv.	Flagged panicle; Glabrous and black lemma
14	*semiglabra* Malz.	Flagged panicle; Hairiness of awn insertion;
15	*trichophora* Malz.	Flagged panicle; Pubescent lemma
16	*secunda* Mordv.	Unilateral panicle, Glabrous lemma
17	*nudibrevis* Vav.	Grains naked

### Morphology

Morphological evaluation was carried out in experimental fields of the Plant Breeding and Acclimatization Institute – National Research Institute in 1980’s. Thirty-six traits (Table 2) were observed similarly as described by W Podyma, M Boczkowska, B Wolko and DF Dostatny [14].

### Isoenzymes

Twelve isoenzymatic systems were tested (Table 3). Isozymes were extracted from two-weeks old seedlings. The biochemical analysis were conducted on five plantlets representing each accession according to the procedure fully described by Podyma et al. [14].

### SRAP

DNA was extracted from leaf tissue of two-weeks old seedlings. Each accession was represented by bulk sample composed of 12 randomly chosen individuals. The tissue was lyophilized and homogenised in bead mill MM301 (Retch). The total DNA was extracted using Genomic Mini AX Plant (A & A Biotechnology). Sixty-four SRAP primers combination were initially tested [54]. Out of them eight the most polymorphic pairs were selected for the further analysis (Table 4). PCR reaction was carried out in 25 μl volume of mixture containing 50 ng DNA, 1u DSF-Taq DNA Polymerase (Bioron), 1x complete KCl reaction buffer containing 15 mM MgCl_2_, 1.2 mM of each dNTP and 0.2 μM of each primer. The PCR amplification was performed using Verity 96 Thermal Cycler (Applied Biotechnology) under following temperature profile: 3 min. at 94 °C followed by five cycles each one including 1 min at 94 °C, 1 min at 35 °C and 1 min at 72 °C, followed by 40 cycles of 1 min at 94 °C, 1 min at 50 °C and 1 min at 72 °C and the final extension for 10 min at 72 °C. Four forward Me primers were labelled at the 5′ end with one of fluorochromes (6-FAM, VIC, NED and PET). The amplified fragments were analysed using capillary sequencer Genetic Analyser 3130XL. The 36 cm capillary array field with NanoPOP7 (Nimagen) was used. The length of fragments was assessed against the GeneScan 1200 LIZ Size Standard (Applied Biosystem). Each PCR reaction and fragment analysis were performed in three independent replicates. Only repeatable fragments were scored.

### Data analysis

SRAP and isoenzymatic fragments were scored and coded as 0/1 matrices, where 0 indicated absence and 1 presence of fragment. Genetic distance was calculated based on Dice formula. The resulting matrices were used in Principal Coordinate Analysis (PCoA). Multiple Factor Analysis (MFA) was performed to simultaneous analysis of qualitative and quantitative morphological traits. Generalized Procrustes Analysis (GPA) was used to reduce the scale effect and to obtained consensus configuration of morphological, izoenzymatic and genetic results. The GPA analysis unifies individual systems or geometric representations on a plane by transforming them through iterative algebraic steps i.e. rotation, translation, reflection and scaling. The transformations must meet two assumptions, i.e. to maintain a relative distance between the elements of each system and to minimize the sum of squares between points which, in different systems, correspond to the same elements [61]. The Pearson correlation was calculated for GPA and geographic coordinates.

The marker informativeness was determined by the Polymorphic Information Content (PIC) coefficient using the following formula:

$$ PIC=1-\sum \limits_{i=1}^n{p}_i^2 $$

where *i* is the *i*^*th*^ allele of the *j*^*th*^ marker, *n* is the number of alleles of the *j*^*th*^ marker and *p* is an allele frequency.

The Shannon-Weaver (H′) index was used as a diversity index and was calculated as follows:

$$ H=-\sum \limits_{i=1}^n{p}_i\ln \left({p}_i\right) $$

$$ {H}_{max}=\ln (n) $$

$$ {H}^{\prime }=\frac{H}{H_{max}} $$

where *H′* is standardized relative diversity index, *n* is the number of classes per trait/marker, *p*_*i*_ is the proportion of the total number of entries in the *i*^*th*^ class. The variation coefficient defined as:

$$ cv=\frac{\sigma }{\mu } $$

where *σ* is the standard deviation and *μ* is arithmetic mean, was used to measure the dispersion of quantitative traits. The unbiased genetic diversity coefficient was calculated as follows:

$$ uGD=\frac{n}{n-1}\left(1-\sum \limits_{i=1}^n{p}_i^2\right) $$

where *n* is the sample size and *p*_*i*_ is the frequency of the *i*^*th*^ trait/marker.

The isoenzymatic and genetic data were subjected to a hierarchical analysis of molecular variance (AMOVA) described by Excoffier et al. [62].

All analyses were performed using the Microsoft Excel 2016, XLSTAT Ecology (Addinsoft, Inc., Brooklyn, NY, USA) and GenAlEx 6.501 [63].

The Bayesian model-based analysis of population structure was performed with the most commonly used software STRUCTURE 2.3.4 [64]. The number of clusters was inferred using ten independent runs with 100,000 iterations and a burn-in period of 100,000 with K values ranging from one to ten. The population structure was analysed assuming admixture in the population in correlated allele frequency model. CLUMPAK software [65] was used to determine the number of true clusters in the data (K). The optimal K was identified based on the posterior probability of the data for a given K, and the ΔK [66]. The best alignment to the replicated results of the cluster analysis was performed with full search algorithm.

## References

[1] Suttie J, Reynolds S (2004). Fodder oats: a world overview. Plant production and protection series, vol. 33.

[2] Kropac Z (1981). *Avena strigosa*: a disappearing synanthropic species in Czechoslovakia. Preslia.

[3] Fernandez MR, dos Santos HP (1992). Contribution of *Avena* spp., used in crop rotation systems under conservation tillage, to the inoculum levels of some cereal pathogens. Can J Plant Pathol.

[4] Steinberg JG, Fetch JM, Fetch T (2005). Evaluation of *Avena* spp. accessions for resistance to oat stem rust. Plant Dis.

[5] Rayapati P, Portyanko V, Lee M (1994). Placement of loci for avenins and resistance to *Puccinia coronata* to a common linkage group in *Avena strigosa*. Genome.

[6] Gregory JW, Wise RP (1994). Linkage of genes conferring specific resistance to oat crown rust in diploid *Avena*. Genome.

[7] Smitterberg M (2018). Differences among variety samples of *Avena strigosa* regarding β-glucan, tocopherols, tocotrienols and avenanthramides.

[8] Kubiak K (2009). Genetic diversity of Avena strigosa Schreb. Ecotypes on the basis of isoenzyme markers. Biodivers Res Conserv.

[9] Weibull J, Bojesen L, Rasomavieius V. *Avena strigosa* in Denmark and Lithuania: prospects for *in situ* conservation. Plant Genet Resour Newsl. 2002;131:1-6.

[10] Rhodes L, Bradley I, Maxted N (2016). Avena strigosa. *The IUCN Red List of Threatened Species 2016*.

[11] Food and Agriculture Organization of the United Nations: World Information and Early Warning System (WIEWS) on Plant Genetic Resources for Food and Agriculture (PGRFA). In*.*; 2018.

[12] Antony T (2007). Evaluation of black oat (*Avena strigosa* Schreb.) germplasm.

[13] Da-Silva P, Milach S, Tisian L (2011). Transferability and utility of white oat (*Avena sativa*) microsatellite markers for genetic studies in black oat (*Avena strigosa*). Genet Mol Res.

[14] Podyma Wiesław, Boczkowska Maja, Wolko Bogdan, Dostatny Denise F. (2016). Morphological, isoenzymatic and ISSRs-based description of diversity of eight sand oat (Avena strigosa Schreb.) landraces. Genetic Resources and Crop Evolution.

[15] Podyma W. Wystepowanie gatunku *Avena strigosa* Schreb. sensu lato oraz zmienosc cech morfologieznych I biochemicznych w populacjach tego gatunku (Distribution of *Avena strigosa* Schreb. sensu lato and morphological and biochemical differentiation within the genus) PhD thesis. Blonie: IHAR-PIB; 1994.

[16] Li G, Quiros CF (2001). Sequence-related amplified polymorphism (SRAP), a new marker system based on a simple PCR reaction: its application to mapping and gene tagging in *Brassica*. Theor Appl Genet.

[17] Budak H, Shearman R, Parmaksiz I, Dweikat I (2004). Comparative analysis of seeded and vegetative biotype buffalograsses based on phylogenetic relationship using ISSRs, SSRs, RAPDs, and SRAPs. Theor Appl Genet.

[18] Robarts DW, Wolfe AD (2014). Sequence-related amplified polymorphism (SRAP) markers: a potential resource for studies in plant molecular biology. Appl Plant Sci.

[19] Ferriol M, Pico B, de Cordova PF, Nuez F (2004). Molecular diversity of a germplasm collection of squash (*Cucurbita moschata*) determined by SRAP and AFLP markers. Crop Sci.

[20] Ferriol M, Pico B, Nuez F (2003). Genetic diversity of a germplasm collection of *Cucurbita pepo* using SRAP and AFLP markers. Theor Appl Genet.

[21] Ferriol M, Picó B, Nuez F (2004). Morphological and molecular diversity of a collection of *Cucurbita maxima* landraces. J Am Soc Hortic Sci.

[22] He F, Yang Z, Zhang Z, Wang G, Wang J (2007). Genetic diversity analysis of potato germplasm by SRAP makers. J Agric Biotechnol.

[23] Chen Y, Li G, Wang X-L (2010). Genetic diversity of a germplasm collection of *Cucumis melo* L. using SRAP markers. Hereditas.

[24] Amar MH, Biswas MK, Zhang Z, Guo W-W (2011). Exploitation of SSR, SRAP and CAPS-SNP markers for genetic diversity of *Citrus* germplasm collection. Sci Hortic.

[25] Khoury C, Laliberté B, Guarino L (2010). Trends in *ex situ* conservation of plant genetic resources: a review of global crop and regional conservation strategies. Genet Resour Crop Evol.

[26] Boczkowska M, Zebrowski J, Nowosielski J, Kordulasińska I, Nowosielska D, Podyma W (2017). Environmentally-related genotypic, phenotypic and metabolic diversity of oat (*Avena sativa* L.) landraces based on 67 polish accessions. Genet Resour Crop Evol.

[27] Rutkoski J, Singh R, Huerta-Espino J, Bhavani S, Poland J, Jannink J, Sorrells M. Efficient use of historical data for genomic selection: a case study of stem rust resistance in wheat. Plant Genome. 2015;8(1):1-10. 10.3835/plantgenome2014.09.0046 33228293

[28] Dawson JC, Endelman JB, Heslot N, Crossa J, Poland J, Dreisigacker S, Manès Y, Sorrells ME, Jannink J-L (2013). The use of unbalanced historical data for genomic selection in an international wheat breeding program. Field Crop Res.

[29] Pozniak C, Clarke J, Clarke F (2012). Potential for detection of marker–trait associations in durum wheat using unbalanced, historical phenotypic datasets. Mol Breed.

[30] Matthies IE, Malosetti M, Röder MS, van Eeuwijk F (2014). Genome-wide association mapping for kernel and malting quality traits using historical European barley records. PLoS One.

[31] International Board for Plant Genetic Resources: Oat descriptors; 1985.

[32] Boczkowska M, Tarczyk E (2013). Genetic diversity among polish landraces of common oat (*Avena sativa* L.). Genet Resour Crop Evol.

[33] Guzmán C, Caballero L, Alvarez JB (2009). Variation in Spanish cultivated einkorn wheat (*Triticum monococcum* L. ssp. *monococcum*) as determined by morphological traits and waxy proteins. Genet Resour Crop Evol.

[34] Coffman Franklin A. (1961). Literature Citations. Oats and Oat Improvement.

[35] Plourde A (1985). Effect of lemma colour on grain quality in oats, *Avena sativa* L.

[36] Holden J: Oats: *Avena* spp. (*Gramineae-Aveneae)*; 1976.

[37] Surje DT, De DK (2014). Correlation coefficient study in oat (*Avena sativa* L.) genotypes for fodder and grain yield characters. J Agric Sci Technol.

[38] Brunava L, Jansone Z, Alsiņa I (2015). Grain yield and its forming parameters variations of oat cultivars. Acta Biol Univ Daugavp.

[39] Linnabary R, Henton J, Heltf J, Black R (1986). Oral ulcerations in a horse caused by grass awns. J Equine Vet Sci.

[40] Mohammadi G, Sardari K (2010). An outbreak of ulcerative stomatitis due to mouse barely (*Hordeum murinum*) in horse. Iran J Vet Sci Technol.

[41] Stace CA. Plant taxonomy and biosystematics. Cambridge: Cambridge University Press; 1991.

[42] Soltis DE, Soltis PS (1990). Isozymes in plant biology.

[43] Phillips T, Murphy J, Goodman M (1993). Isozyme variation in germplasm accessions of the wild oat *Avena sterilis* L. Theor Appl Genet.

[44] Murphy JP, Phillips T (1993). Isozyme variation in cultivated oat and its progenitor species *Avena sterilis* L. Crop Sci.

[45] Nevo E, Beiles A, Zohary D (1986). Genetic resources of wild barley in the near east: structure, evolution and application in breeding. Biol J Linn Soc Lond.

[46] Marshall D, Brown A. Wheat genetic resources. Cambridge: Cambridge Univ. Press; 1981.

[47] Volis S, Mendlinger S, Turuspekov Y, Esnazarov U (2002). Phenotypic and allozyme variation in Mediterranean and desert populations of wild barley, *Hordeum spontaneum* Koch. Evolution.

[48] Souza E, Sorrells M (1989). Inheritance and frequency of a null allele for diaphorase activity in north American oat cultivars. J Hered.

[49] Kanwal K, Singh R, Singh J (1983). Divergent gene pools in rice improvement. Theor Appl Genet.

[50] Reed DH, Frankham R (2001). How closely correlated are molecular and quantitative measures of genetic variation? A meta-analysis. Evolution.

[51] Price S, Shumaker K, Kahler A, Allard R, Hill J (1984). Estimates of population differentiation obtained from enzyme polymorphisms and quantitative characters. J. Hered..

[52] Al-Kaab DH, Hamdalla MS, Dweikat IM, Al-Saedi NJ (2016). Estimation of the degree of diversity for some Iraqi wheat varieties through ISSR, SRAP and RAPD markers. Am J Exp Agric.

[53] Zheng Y, Xu S, Liu J, Zhao Y, Liu J (2017). Genetic diversity and population structure of Chinese natural bermudagrass [*Cynodon dactylon* (L.) Pers.] germplasm based on SRAP markers. PLoS One.

[54] Budak H, Shearman R, Parmaksiz I, Gaussoin R, Riordan T, Dweikat I (2004). Molecular characterization of buffalograss germplasm using sequence-related amplified polymorphism markers. Theor Appl Genet.

[55] Cheng Y, Ma X, Zhou K, Humphreys MW, Zhang XQ (2016). Phylogenetic analysis of *Festuca–Lolium* complex using SRAP markers. Genet Resour Crop Evol.

[56] Hunter H (1924). Oats; their varieties and characteristics: a practical handbook for farmers, seedsmen, and students.

[57] Loskutov I (2007). Oat (*Avena* L.) distribution, taxonomy, evolution and breeding value.

[58] Loskutov Igor G., Rines Howard W. (2011). Avena. Wild Crop Relatives: Genomic and Breeding Resources.

[59] Federizzi L, Mundstock C (2004). Fodder oats: an overview for South America. Fodder oats: a world overview plant production and protection series.

[60] Rodionova N, Soldatov V, Merezhko V, Jarosh N, Kobyljanskij V (1994). Flora of cultivated plants.

[61] Gower JC (1975). Generalized procrustes analysis. Psychometrika.

[62] Excoffier L, Smouse PE, Quattro JM (1992). Analysis of molecular variance inferred from metric distances among DNA haplotypes - application to human mitochondrial-DNA restriction data. Genetics.

[63] Peakall R, Smouse PE (2012). GenAlEx 6.5: genetic analysis in excel. Population genetic software for teaching and research-an update. Bioinformatics.

[64] Pritchard JK, Stephens M, Donnelly P (2000). Inference of population structure using multilocus genotype data. Genetics.

[65] Kopelman NM, Mayzel J, Jakobsson M, Rosenberg NA, Mayrose I (2015). Clumpak: a program for identifying clustering modes and packaging population structure inferences across K. Mol Ecol Resour.

[66] Evanno G, Regnaut S, Goudet J (2005). Detecting the number of clusters of individuals using the software STRUCTURE: a simulation study. Mol Ecol.

